# Structural Preservation Percutaneous Endoscopic Lumbar Interlaminar Discectomy for L5-S1 Herniated Nucleus Pulposus

**DOI:** 10.1155/2016/6250247

**Published:** 2016-10-10

**Authors:** Jung-Sup Lee, Hyeun-Sung Kim, Jee-Soo Jang, Il-Tae Jang

**Affiliations:** ^1^Department of Neurosurgery, Nanoori Suwon Hospital, 295 Jungbu-daero, Yeongtong-gu, Suwon-si, Gyeonggi-do, Republic of Korea; ^2^Department of Neurosurgery, Nanoori Seoul Hospital, 731 Eonju-ro, Gangnam-gu, Seoul, Republic of Korea

## Abstract

*Objective*. Structures such as ligamentum flavum, annulus, and lamina play an important role in the segmental function. We proposed the surgical technique for achieving the sufficient preservation of segmental structures, in spite of sufficient removal of pathologic disc in the L5-S1 using the ligamentum flavum splitting and sealing technique.* Methods*. We retrospectively analyzed 80 cases that underwent percutaneous endoscopic lumbar discectomy for L5-S1 herniated nucleus pulposus, using the ligamentum flavum splitting and sealing technique between January 2011 and June 2013. Outcomes were assessed using VAS (leg, back), MacNab's criteria, and the immediate postoperative MRI for all patients. Structural preservation was classified as complete, sufficient, and incomplete.* Results*. The surgical results are as follows: 65 cases were complete, 15 cases were sufficient, and 0 cases were incomplete. The VAS was decreased at the last follow-up (leg: from 7.91 ± 0.73 to 1.15 ± 0.62; back: from 5.15 ± 0.71 to 1.19 ± 0.75). A favorable outcome (excellent or good outcome by MacNab's criteria) was achieved in 77 patients (96.25%). During the follow-up period, 2 cases (2.5%) of recurrence have occurred.* Conclusion*. According to the result, we could obtain the favorable clinical and radiological outcomes while simultaneously removing pathologic discs using the ligamentum flavum splitting and annular fissure sealing technique.

## 1. Introduction

Endoscopic disc surgery has made great progress since it was introduced in the 1980s [1]. Its most important goal is to provide minimally invasive approaches in herniated disc surgery. Ideally, the ultimate goal of endoscopic discectomy is to achieve outcomes similar to or better than those of conventional open discectomy. Endoscopic discectomy has advantages in providing clear visualization of normal and pathological tissues and minimizing damage to adjacent tissue, with preservation of normal structures. However, it has the disadvantage of a relatively longer learning curve in comparison with conventional open surgery [2, 3].

Transforaminal and interlaminar approaches are mainly used in endoscopic lumbar discectomy [4]. The transforaminal approach shows good results for nerve root decompression, with low complication rates in herniated lumbar disc removal, and therefore has been considered a standard procedure at lumbar spine [4–8]. However, at the L5-S1 level, the iliac crest often blocks the trajectory that approaches the foramen, and the L5 transverse process and hypertrophied facet joints can often hinder the procedure; if a transforaminal approach is difficult, a transiliac approach can be used, but the interlaminar approach is preferred at the L5-S1 level [4, 8–11].

An endoscopic interlaminar approach at the L5-S1 level was introduced in 2006 and has been used by many surgeons since, with techniques gradually being refined [9, 10, 12–14]. Managing the ligamentum flavum is often problematic in an L5-S1 interlaminar approach, which requires cutting or splitting. The ligamentum flavum plays an important role in postoperative scar formation, and annular preservation is also important for structural maintenance and prevention of recurrence [15, 16]. This study aimed to remove only the pathological disc while maintaining the surrounding structure as much as possible: first, the ligamentum flavum was split under direct visual control; next, annular fissure sealing with coagulation was performed for annular restoration and to prevent recurrence. The purpose of this study was to introduce the techniques and report our experience.

## 2. Materials and Methods

### 2.1. Patients and Characteristics

A retrospective analysis was conducted on eighty patients (male : female = 51 : 29) (Table 1) who underwent endoscopic lumbar interlaminar discectomy for a symptomatic L5-S1 herniated lumbar disc between January 2011 and June 2013. The ligamentum flavum splitting and annular fissure sealing technique were implemented on all patients. The surgical indications were unilateral radicular leg pain unresponsive to conservative treatments and posterolateral ruptured disc herniation at L5-S1 level confirmed by magnetic resonance imaging (MRI) corresponding to clinical symptoms. All patients underwent either sequestrectomy or fragmentectomy. Patients who needed extensive discectomy due to stenosis or calcified discs were excluded. Recurrence at L5-S1 level was also excluded along with other pathology, such as infection, tumor, and fracture.

This study was approved by the Institutional Review Board of Nanoori Hospital (NR-IRB 2016-002).

### 2.2. Surgical Technique

**Figure 1:**
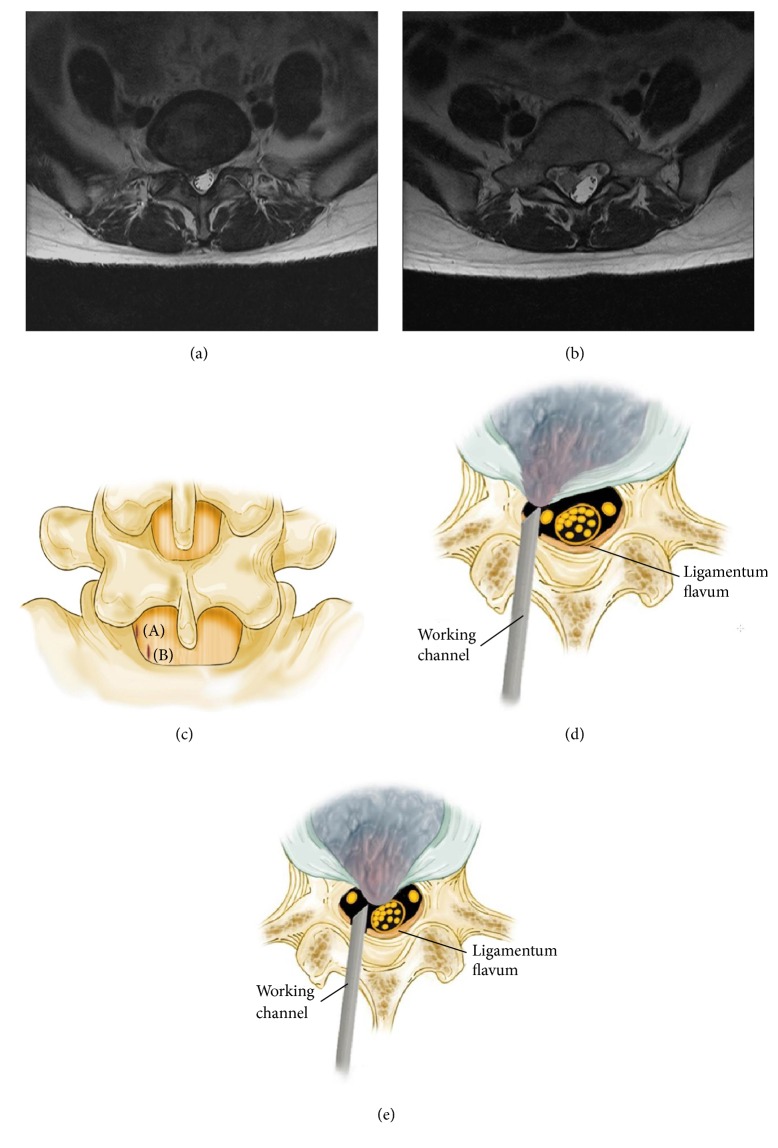
Surgical procedures. (a) Preoperative T2-weighted magnetic resonance image (MRI) shows the shoulder type disc herniation. (b) Preoperative T2-weighted MRI shows the axillar type disc herniation. (c) Ligamentum flavum vertical splitting performed using a dissector more laterally in shoulder type (A) and more caudally in axillar type (B). (d) and (e) Introduced the working channel bevel into the splitting area and rotated to widen the opening. And then we retract the thecal sac or root using working channel bevel in shoulder type (d) and axillar type (e).

**Figure 2:**
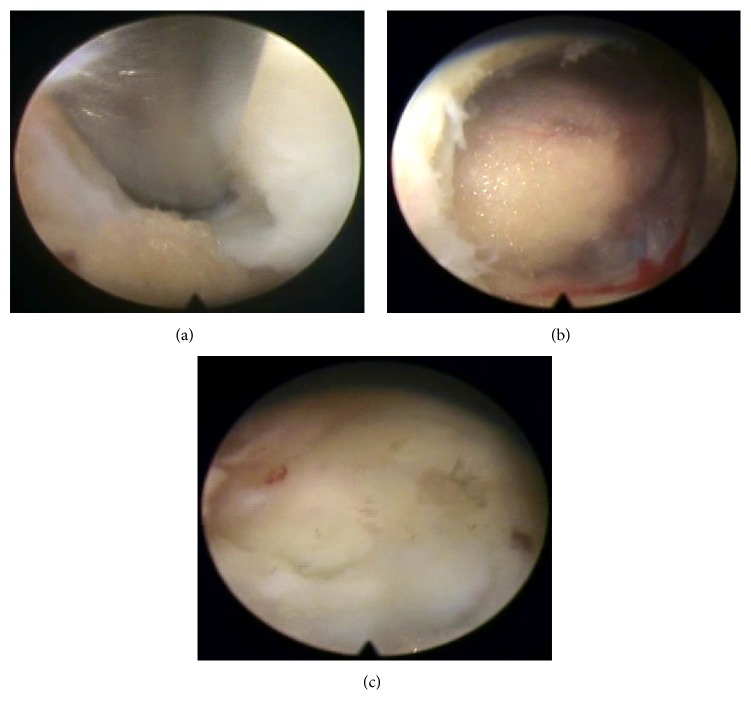
Intraoperative endoscopic view showing the ligamentum flavum. (a) Ligamentum flavum splitting using dissector in vertical direction. (b) Rotating and introducing the working channel bevel into the epidural space. (c) After the operation, closed ligamentum flavum splitting line was seen when the working channel was removed.

**Figure 3:**
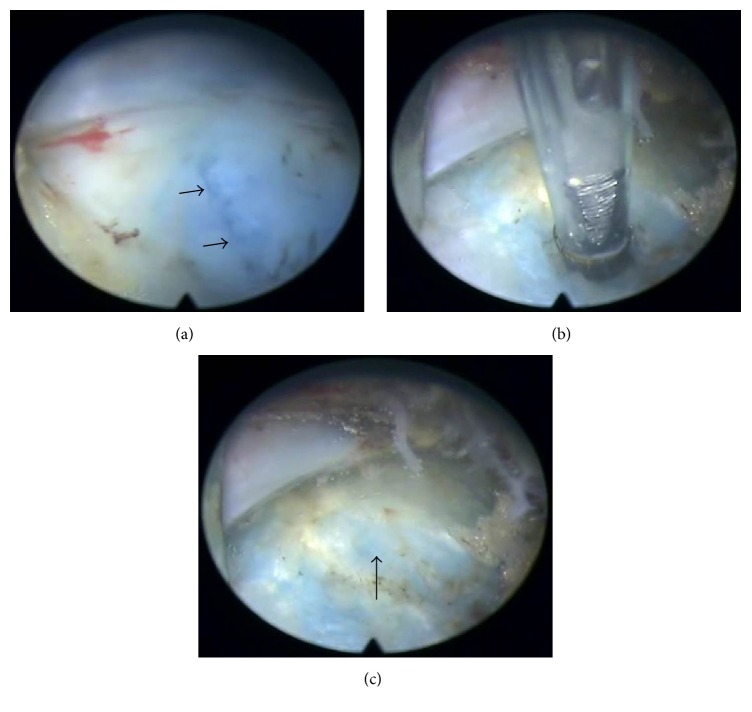
Images during the endoscopic discectomy with annular sealing. (a) Before annular sealing, annular defect was seen (black arrow). (b) Annular sealing using bipolar coagulation. (c) After annular sealing, the annular defect became smaller (black arrow).

Surgery was conducted under epidural anesthesia using C-arm fluoroscopic guidance. The endoscopic interlaminar discectomy method at L5-S1 level starts with the same method as introduced before but is slightly modified for ligamentum flavum management. Previously, a small hole was made in the ligamentum flavum or sequential insertion of serial dilators in the ligamentum flavum was performed for an approach through the epidural space; the present technique splits and widens ligamentum flavum using a dissector and working channel. The greatest advantages are that a hole is not created in the ligamentum flavum by excision, and there is simultaneous visual verification through the endoscopic view. First, under epidural block, discography was performed with indigo carmine mixed with radiopaque dye, using a transforaminal approach at L5-S1, and then an obturator and working channel were advanced to the ligamentum flavum in the interlaminar space. After endoscope insertion, obstructing muscle and fat were removed to visualize the ligamentum flavum. Vertical splitting was then performed using a dissector, and the bevel of a working channel was inserted into the split ligamentum flavum and rotated to widen the opening, while simultaneously entering the epidural space. Once in the epidural space, fat tissue and S1 roots can be visually verified, and sometimes extruded disc can be seen (Figures 1, 2(a), and 2(b)). In accordance with the disc location confirmed on MRI, the thecal sac and the root were controlled using the bevel of a working channel and sequestrectomy or fragmentectomy was performed. Usually an annular cutter or punch can be used to widen the disc annulus fissure. But we used a dissector to minimize the size of the annulus fissure as much as possible. After sequestrectomy or fragmentectomy, annulus fissure coagulation was performed using bipolar radiofrequency (Elliquence, LLC, Trigger-Flex®). The power was set at 15 W for coagulation, which was performed along the margins of the fissure, sweeping from the edge toward the center; we were able to visually verify decreasing fissure size as the margins of the fissure were constricted in the endoscopic view (Figure 3). Before completing the surgery, it is important to reconfirm the surroundings of the S1 root area. For example, following the removal of disc fragments in the axillary area of the S1 root, the shoulder area that was not previously visible needs to be checked for any remaining disc fragments using the bevel of a working channel; if the shoulder region was treated first, the axillary area should be checked.

### 2.3. Outcome Assessment

**Figure 4:**
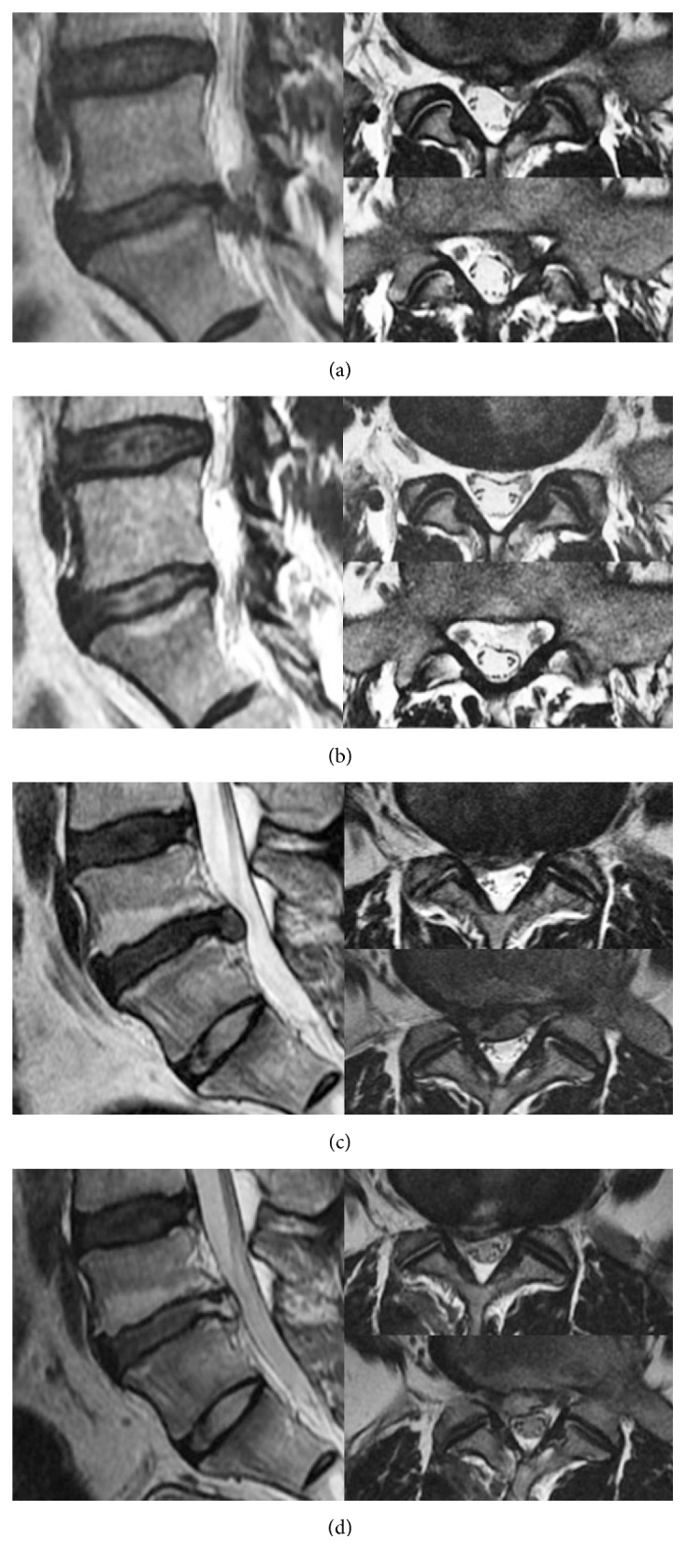
Radiological results using MRI. (a) and (b) Categorized as complete. Pre- and postoperative MRI showing the preserved ligamentum flavum, complete pathologic disc removal, and complete annulus restoration. (c) and (d) Categorized as sufficient. Pre- and postoperative MRI showing the preserved ligamentum flavum, complete pathologic disc removal, and incomplete annulus restoration.

Clinical results were assessed using the following instruments: a visual analog scale (leg and back VAS, scored 1–10) and MacNab's criteria. Radiological results were assessed using an immediate postoperative MRI that was performed to verify structural preservation. MRI was categorized by operator and checked by another two spinal neurosurgeons who expertise in the percutaneous endoscopic spine surgery. The classification was categorized as complete (preserved ligamentum flavum + complete pathologic disc removal + complete annulus restoration), sufficient (preserved ligamentum flavum + complete pathologic disc removal + incomplete annulus restoration), or incomplete (ligamentum flavum not preserved + incomplete pathologic disc removal + incomplete annulus restoration) (Table 2) (Figure 4). Statistical analysis was performed using SPSS version 20.0 (SPSS, Chicago, IL, USA). Independent *t*-test and Chi-square test were used to determine the differences between recurrence and all other parameters. All *p* values less than 0.05 were considered statistically significant.

## 3. Results

The mean age of the patients in this study was 40 ± 12 years (range 18 to 73), and the mean follow-up period was 13 ± 6 months (range 6 to 28). The symptoms improved in all patients immediately after surgery, and all were discharged the next day. There were no complications related to the surgery. Radiological verification of structural preservation showed that 65 cases were complete (81.25%) and 15 were sufficient (18.75%); there were no incomplete cases (0%) (Table 3). The mean VAS (leg, back) for clinical results decreased from 7.91 ± 0.73 before surgery to 1.15 ± 0.62 at final follow-up for leg pain, from 5.15 ± 0.71 before surgery to 1.19 ± 0.75 at final follow-up for back pain (Table 4). According to MacNab's criteria, a favorable outcome (excellent or good) was achieved in 77 patients (96.25%) and an unfavorable outcome (fair or poor) was observed in 3 patients (3.75%) (Table 5). During the follow-up period, two cases (2.5%) had recurrence (one case was early, less than 6 months; one case was delayed, more than six months); there were no statistically significant differences between recurrence and age (*p* = 0.645) and sex (*p* = 0.682).

## 4. Discussion

In surgical treatment of lumbar disc herniation, conventional open surgery has been considered the gold standard, but endoscopic discectomy has often been used since the advent of minimally invasive surgery [17]. With advances in endoscopic technique and instrumentation, for example, drills and curved forceps, a wider range of applications has evolved. Many studies have shown good success rates, and some reported superiority compared to conventional discectomy [4–6, 8, 9, 11, 18–26]. Advantages of endoscopic surgery include less trauma to surrounding muscles, ligaments, or facet joints and less scarring, adhesions, and instability after surgery and reduced length of hospital stay and a quick return to normal life [4, 15, 16, 27–30].

Removal of intradiscal nucleus material at the L5-S1 level is limited by the divergence between the disc space and interlaminar window; thus, sequestrectomy or fragmentectomy was performed rather than aggressive discectomy [9, 10, 29, 30]. However, according to many studies comparing sequestrectomy and wide discectomy performed in conventional open surgery, there is little difference in the recurrence rate [31–33]. Furthermore, there have been reports of decreased operative time and decreased intraoperative complications with sequestrectomy and reports of less degenerative change after surgery compared to conventional methods; thus, it can be assumed that sequestrectomy or fragmentectomy is adequate [34, 35].

The most important thing to consider in an interlaminar approach is management of the ligamentum flavum. The transforaminal approach eliminates this concern because it does not go through the ligamentum flavum. However, the interlaminar approach uses the same routes as in conventional discectomy, and the ligamentum flavum must be handled. Previous studies have described three methods for ligamentum flavum management: one is to create a 3 × 5 mm hole as an approach to the epidural space [4]; another is to insert a wire into the disc space under fluoroscopic guidance, while checking the patient's responses, followed by sequential insertion of serial dilators to approach the epidural space [4, 9]; the third combines the benefits of the first two, with ligamentum flavum splitting under direct visual control [36]. The first method is safer, with excision and surgery under visual control after examination of the surface of the ligamentum flavum. However, despite the small hole, injury to the ligamentum flavum cannot be prevented [36]. The second method results in less damage to ligamentum flavum because the operation is performed with the ligamentum flavum splitting. However, it is possible to injure the root and thecal sac since the procedure is not under direct visual control [9]. Previous reports suggest that this approach is safe, but a blind operation always has risk [13, 36].

We used the third method with vertical, linear splitting of the ligamentum flavum using the dissector and then approaching the epidural space using the bevel of a working channel. We anticipated less damage to the ligamentum flavum and less chance of injury to the root or thecal sac, because the approach was performed under visual control and also because no hole was punched in the ligament [36]. Damage to the ligamentum flavum is known to cause scars, and epidural scars reportedly cause postoperative pain [10, 37, 38]. Thus, it is often preferred to leave the ligamentum flavum intact in order to reduce scarring, even in conventional open surgery; efforts to reduce scarring to a minimum include fat harvesting or use of an antiadhesive agent [15, 16, 39, 40]. The ligamentum flavum splitting technique is in line with this trend and is a safe treatment method causing the least damage. In fact, the splitting line can be seen to close well as the working channel is removed (Figure 2(c)).

The second technique we used for structural preservation is annular sealing. This involves coagulation using bipolar radiofrequency in the area surrounding the annular fissure. Tissues shrink due to heat energy, reducing the fissure and tightening the loose annular tissue in the surrounding area [30, 41–43]. The major concern in discectomy, with both conventional open surgery and endoscopic discectomy, is recurrent disc herniation [4, 9, 44, 45]. It is important to reduce defects to the smallest possible size because annular defect size can affect recurrent disc herniation [46–48]. Some operative methods using instruments or suturing are used in conventional open surgery to reduce annular defects, and these methods reportedly decrease the disc reherniation [49]. Furthermore, previous studies on endoscopic surgery have examined the effect of annular sealing, and it is assumed that sealing using bipolar radiofrequency to reduce annular defects can be effective in reducing recurrence rates [30].

It was difficult to verify recurrence because of uncertain criteria. Previous studies cannot be used for comparison because of the differing criteria for recurrence [30]. Recurrence after percutaneous endoscopic lumbar discectomy usually occurred within 6 months [10, 44, 45]. In the percutaneous endoscopic lumbar discectomy, early recurrence will be an important issue of recurrence. According to the report by Kim et al., they defined early reoperation as the reoperation within 90 days of initial operation [50]. Therefore, in this study, we enrolled the patients with more than 6 months of follow-up and recurrence was defined as cases of disc herniation at the same level after a pain-free postoperative interval. There are few reports about the recurrence rates after percutaneous endoscopic interlaminar lumbar discectomy (range 0.1–6.6%) and conventional sequestrectomy (range 2–18%) [30]. The recurrence rate in this study was approximately 2.5%, which is similar to or slightly better than the recurrence rate in previous percutaneous endoscopic interlaminar lumbar discectomy and conventional sequestrectomy studies. However, a consensus on criteria for recurrence is necessary, including the criterion we used.

### 4.1. Limitation

There are limitations in this retrospective study. In order to verify the usefulness of structural preservation with ligamentum flavum splitting and annular fissure sealing techniques, prospective studies are needed. Also, this is not a comparative study but only shows results. Furthermore, this is a short-term follow-up study, and long term follow-up and further studies are needed; however, these are not difficult or lengthy procedures. It is clear that structural preservation is an important component of endoscopic surgery, and we hope that these techniques will be shared. Despite the limitations, the study outcomes show the usefulness of these techniques.

## 5. Conclusion

The results show a favorable clinical outcome using structural preservation techniques for endoscopic interlaminar lumbar discectomy of L5-S1 herniated discs. A combination of ligamentum flavum splitting and annular fissure sealing techniques is useful in preserving the annulus and associated structures, while simultaneously removing the pathologic disc.

## Figures and Tables

**Table 1:** Demographic features of the 80 patients who underwent PEID with ligamentum flavum splitting and annular sealing.

Characteristics	Number of patients (*N* = 80)	%
Sex		
Male	51	64
Female	29	36
Age, years		
<30	16	20
31–40	27	34
41–50	21	25
51–60	12	15
61–70	2	3
>71	2	3

**Table 2:** Radiological results.

Radiologic results	Ligamentum flavum	Pathologic disc removal	Annulus restoration
Complete	Save	Complete	Complete
Sufficient	Save	Complete	Incomplete
Incomplete	No save	Incomplete	Incomplete

**Table 3:** Radiological results.

Radiologic results	Number of patients (*N* = 80)	%
Complete	65	81.25
Sufficient	15	18.75
Incomplete	0	0

**Table 4:** Clinical results—VAS.

	Preoperative		Postoperative		*p* value
	Mean	(±SD)	Mean	(±SD)	
VAS (leg)	7.91	0.73	1.15	0.62	<0.001
VAS (back)	5.15	0.71	1.19	0.75	<0.001

**Table 5:** Clinical results—MacNab's criteria.

	*N*	%
Poor	0	0.0
Fair	3	3.75
Good	13	16.25
Excellent	64	80.0
